# DFT analogue of prospecting the spin-polarised properties of layered perovskites Ba_2_ErNbO_6_ and Ba_2_TmNbO_6_ influenced by electronic structure

**DOI:** 10.1038/s41598-022-22070-x

**Published:** 2022-11-16

**Authors:** Saveer Ahmad Khandy, Dinesh C. Gupta

**Affiliations:** Condensed Matter Theory Group, School of Studies in Physics, Jiwaji University, Gwalior, 474011 India

**Keywords:** Materials science, Physics

## Abstract

Since the unexpected accelerated discovery of half-metallic perovskites is continuously on the rise both from basic sciences and application-oriented sides. Herein, for the first time in this carried research work, we significantly delivered a detailed analysis on one of experimentally synthesized perovskite structure Ba_2_ErNbO_6_ and in related to Ba_2_TmNbO_6_ within the realm of unified density functional theory. Initially, the structural stability of two molecular perovskite structures were critically established interms of their total ground state and cohesive energies by the expendition of Brich Murnaghan equation of state. Also, the tolerance factor (τ) oversees the cubic structural stability without possessing any geometrical strains. More likely, the density functional perturbation theory (DFPT) has been calibrated to perceive the dynamical context of these layered structures. Also, from the understandings of second order elastic and mechanical parameters adresses their suitable ductile characteristics. The quantum mechanical refinement of their intrinsic electronic structures were systematically tuned by the exploitation of Generalised gradient approximation (GGA), on-site Hubbard scheme (GGA + U) selected to the strongly correlated electrons of particular angular momentum and modified Becke-Johnson (mBJ) potential. Moreover, the two-dimensional representation of asymmetric density of states (DOS) pinned around the Fermi-level (E_F_) and the interpretation linked to their corresponding spin-polarised band structures signatures the well-known half-metallic nature. Subsequently, the transport properties especially the value of figure of merit (_Z_T) equals to unity (1) along the selected chemical potential range at different temperatures. The summed-up properties and the overall tendency triggers the possibility of these materials to register their extending applications in spintronics, thermoelectrics, nanoengineering, and radioisotope generator perspectives.

## Introduction

The search of novel half-metallic double perovskites having their relaxed flexibility and tunability has turned a momentous gear by extending their advantages in various hotspots of research. Half-metallic double perovskites offer a resurgence of interest and thus became a point of prestige for their outstanding multidimensional and multifunctional tendencies which lends them striking applicants in near future advancements^1–3^. Keeping in view, this unified class has been steadily and widely anticipated, significantly developing a central theme till date now and alot accelerated work either experimentally or theoretically has been enormously laid to snatch their fundamental properties which exactly fits in energy harvesting technologies^4,5^. As it is clear now, that global energy crises and environmental deterioration is a critical issue due to the vast consumption and contamination of energy resources^6–11^. Also, the tremendous exploitation of fossil fuels are now at the verge of extinction. The overwhelming and unprecedented use of natural reservoirs and fluctuating prices of energy products creates a big threat for surviving the sustainable life. Consequently, the worldwide energy demand and the substantial need to run the daily life activities crucially needs some orthodoxies to resolve such an expansionary problem^12^. The researchers all over the world are consistent to find the new alternatives to overcome such issues. However, solid-state materials among the prestigious oxide perovskites demonstrating the spectacular performance by showing the thermoelectric behavior with the working principle of Seebeck effect. The efficiency of a thermoelectric material can be configured by the mathematical formula;

1 $$ ZT = \frac{{S^{2} \sigma T}}{{\kappa_{e} + \kappa_{l} }} $$

The terms in the benchmark studies has been described by others^13–15^. However, for a good thermoelectric material, it should obey many phenomenological requirements for bestowing good figure of merit (zT) if its Seebeck coefficient, electronic conductivity is compensatively large, but could have lower value of thermal conductivity. A material with *zT* ~ 1.0 is considered to be a good thermoelectric material^16^. In the recent past, several double perovskites has been exposed as efficient thermoelectric materials featuring *zT* = 1^17,18^ proposing their advantages for both refrigeration and power generation applications. Also, the results confirm some of the earlier ideas of Dresselhaus and collaborators suggested that nanoengineering of thermoelectric materials could result in decent values of *zT*^19^. Subsequently, actinide-based double perovskites for their extending applications in radioisotope generator applications (RTG’s)^20,21^ have received a dignified significance. Remarkably, the exhibition of intriguing electronic structures especially the character of owing half-metallic nature descripts 100% spin-polarisation inclines their facial route towards the significant perspectives in spintronics, spin dynamics^22,23^ etc. Apparently, with such spell bound applications, various double perovskites in the aforementioned studies has been reported as half-metallic compounds and are compatible for conventional spin-based technologies^24–26^. Besides this, the main strategy of exploring these double perovskites is to determine the fruitful applications to tackle the present situations.Tracking down to the ground information of these present perovskites. The information perceived from one of the material (Ba_2_ErNbO_6_) was experimentally synthesized^27,28^. The procedure followed by several authors exclusively claims the cubic crystalline nature of this perovskite oxide. On the otherhand, a literature survey of Ba_2_TmNbO_6_ has been mentioned in benchmark studies^29^. Moreover, on this basis of their reported information, we have tried to describe the properties of these materials intently by density functional theory (DFT) method. The method of applying such simulation is quite advanced in describing the physical properties which potentially facilitates their extending application stand towards spintronics and energy harvesting technologies.

## Computational aspects (theoretical research approach)

The calculations has been precisely carried to descript the applicability of Ba_2_ErNbO_6_ and Ba_2_TmNbO_6_ layered structures in compatible technologies such as spintronics in solid state device applications and thermoelectrics for power generation sources. Thereefore, the computation of doing this research has been consistently followed by solving the Kohn–Sham equations within the domains of density function theory (DFT) as compressed in *Wien2k*^30^. In the beginning, several functional schemes viz, GGA^31^, on-site GGA + U^32–35^ and later on GGA + mBJ^36–39^ has been alternatively acted upon these perovskite systems to execute their electronic structures by testifying their exchange correlation potential (*E*_*xc*_). The exchange correlation is however a question mark in density functional theory while defining the possible electronic profiles of many body systems. So, the first approximation being selected to tackle the exchange correlation potential was generalised gradient approximation (GGA). In GGA formulism, the exchange–correlation (*E*_*xc*_) is viewed as a derivative of the local charge density and the associated gradient. However, from the previous studies it is quite often that the implementation of this scheme specifically for the systems accompanied with *d/f* electrons is incompetent to display the resulting electronic structures considerably leads the discrepancies in magnetic properties as well and hence aids several pitfalls due to the fact of self-interaction error. The self-interaction problem arises from the non-complete cancellation of the spurious repulsion of each electron from itself and the absence of any derivative discontinuity in GGA functional. By staying within DFT, precise electronic structures of these perovskites can also been achieved technically by introducing sophisticated on-site Hubbard parameter (GGA + U) which is fitted in accordance to the total energy convergence. A reasonable U_*eff*_ (U_*eff*_ = U − J), where Coulomb interaction (U) of 0.36 and 0.38 Ry has been adjusted for both Nb-*d* and Er/Tm *f*-states of particular angular momentum respectively and J defines the exchange interaction is kept at zero. Since, the on-site term is applied only inside the sphere surrounding the atom of interest, the results may depend on the radius *R*_MT_ of the sphere which is a drawback of the on-site method. Therefore, the involvement of *d*/*f* electrons invokes or allows us to employ a pure ab initio strategy to refine their electronic structures precisely, known as modified Becke Johnson (mBJ) potential. The exploitation of such systematic potential is referred as a better choice with its accuracy of descripting the results prior to the experimental results. Besides this, the DFT calculations has been further extended by selecting a mesh of 1000 k-points in the momentum space and *R*_MT_*K*_max_ = 7 to run the self-consistent field where the terms (*R*_MT_) defines the smallest muffin-tin radii is used to take care of electron hopping from core to interstitial regions and *K*_max_ explains the plane wave expansion. The numerical energy value of − 7.0 Ry is assigned for core and valence states^40^. The specific values of both charge and energy has been adopted to 10^−4^ a.u.^3^ and 10^−4^ Ry respectively during the whole calculation process and the integrated charge difference between two successive iterations has been taken less than 10^−4^/a.u^3^. In addition, the elastic constants associated with the second-order change of the internal energy has been simulated in view of Cubic elastic code^41^. Moreover, density functional perturbation theory integrated in Quantum espresso^42^ has been decisively used to descript the dynamical stability of these two perovskites. Finally, the use of semiclassical Boltzmann theory within BoltzTraP scheme^43,44^ has been exploited to explore the transport coefficients.

## Results and discussions

The extensive use of proper functionals to define the inherited physical properties influenced by their electronic structures has been keenly estimated during the calculation process and their detailed summary has been systematically discussed under the following subheadings.

### Crystallographic structure and magnetic ground state stability

**Figure 1:**
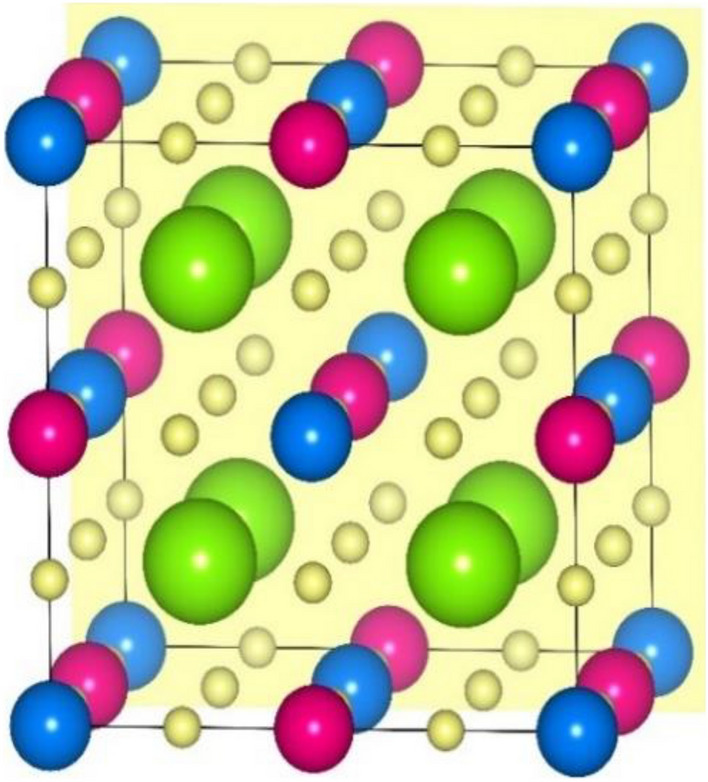
Distinct position of respective atoms of Ba_2_ErNbO_6_ and Ba_2_TmNbO_6_ molecular crystal structures: Green (Ba), Blue (Er/Tm), Pink (Nb) and Yellow (O).

Primarily, the proposed Ba_2_ErNbO_6_ perovskite structure from the rietveld refinement of X-ray diffraction pattern (XRD) correspondingly identifies the face centered cubic (FCC) crystalline structure with along with space orientation *Fm-3m* (225)^27,28^. Therefore, also from the theoretical analysis, which perfectly displays the rational accord with the experimental observation inclines the same cubic structural arrangement of atoms within these kind of systems. So, consistent with this, it is quite interesting to employ a strategy of descripting the various constituent atomic locations within their lattice structures. Interestingly, double perovskites with stochiometric formula A_2_BB′O_6_^45,46^ featured by the ordered distribution at the B site of two different cations showing specific dimensions and charge, typically assuming the rock salt type of ionic packing where the Ba^2+^ atom displays its atomic occupancy at its concerned Wyckoff crystallographic coordinates as per the rule at 8b (0.25, 0.25, 0.25) and (0.75, 0.75,0.75), Er/Tm^+3^ resides at 4a (0, 0, 0), while Nb^+5^ demarcates its location at 4c (0.25, 0.25, 0.25) fixed positions and O^−2^ is embedded at 24e (*x*, 0, 0); *x* = 0.26 where *x* is an internal parameter taken from the predefined experiment. Thus, the structural arrangement of atoms for both these double perovskites can be schematically viewed at Fig. 1.

Moreover, the structural arrangement or occupancy of different atoms at different cites allows us to see the structural stability of Ba_2_ErNbO_6_ and Ba_2_TmNbO_6_ interms of their stable ground state energies. For which the Birch-Murnaghan equation^47^ at the cost of structural optimisation has been put forwarded in distinct ferromagnetic (FM) and non-magnetic (NM) configurations by performing a least-squares fit of the crystal energy against the unit cell volume enumerated by the underneath equation;

2 $$ E\left( V \right) = E_{O} + \frac{{9V_{O} B_{O} }}{16}\left\{ {\left[ {\left( {\frac{{V_{O} }}{V}} \right)^{\frac{2}{3}} - 1} \right]^{3} B_{0}^{\prime } + \left[ {\left( {\frac{{V_{0} }}{V}} \right)^{\frac{2}{3}} - 1} \right]^{2} - 1\left[ {6 - 4\left( {\frac{{V_{O} }}{V}} \right)^{2/3} } \right]} \right\} $$

**Figure 2:**
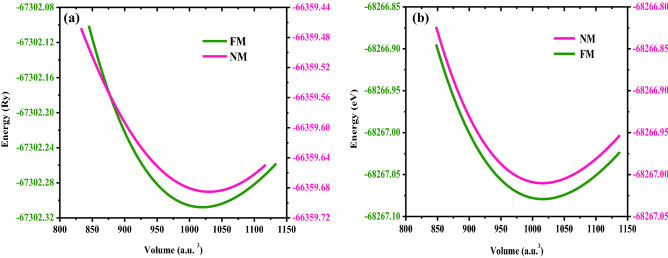
**(a, b)** DFT structural relaxation of (**a**) Ba_2_ErNbO_6_ and (**b**) Ba_2_TmNbO_6_ in spin-polarised (FM) and non-spin polarised (NM) configurations.

The terms *E*(*V)*, *V*, *and* *B*_0_ (*B'*_0_) in this equation connotes the ground state energy, unit cell volume, and the bulk modulus (derivate of bulk modulus), respectively. Basically, the logical explanation of carrying the successful optimisation explains the ground state stability of these two perovskite structures interms of their energies and relies on the justification that the supplementation of the external pressure is responsible for the atoms of their crystal structures to get closer with each other so that the maximum electron density gets acquire which leads to the release of energy. This happens so, because the system always wants to remain at the lower potential. However, the basic principles which involves that when the bond formation takes place, the energy gets released or we can say some sort of exothermic reaction. Therefore, the energy retrieved via from the Brich Murnaghan equation by doing the same procedure in different ferromagnetic (FM) and non-magnetic (NM) configurations of these perovskites certifiy the dominant stability interms of their energies in FM rather than competing NM configurations as portrayed in Fig. 2a,b.

Theoretically speaking, the amount of energy released in FM for Ba_2_ErNbO_6_ and Ba_2_TmNbO_6_ shows the perfect occurrence. Alongside, the total ground state energy of these cystal structures is accompained with other thermodynamical parameters inclusive of volume (***V***_**0**_), bulk modulus (***B***_**0**_**)**, its derivative (***B’***_**0**_**)**, and energy (***E***_**0**_) which has been fetched and enlisted in Table 1. The high value of bulk modulus reflects the rigid nature of these given compounds. Further understanding of ferromagnetic ground state energies can be predicted from their corresponding spin-polarised band structures also.

**Table 1:** Retrieved physical characteristics of Ba_2_ErNbO_6_ and Ba_2_TmNbO_6_ in ferromagnetic (FM) and non-magnetic (NM) configurations.

Parameter	*a*_0_ (Å)			*V*_0_ (a.u.^3^)	*B*_0_ (GPa)	*B′*_0_	*E*_0_ (Ry)	*τ*
	Phase	Present	Others					
Ba_2_ErNbO_6_	FM	8.45	8.30_Expt_^27^ 8.41^28^	1019.60	141.11	4.88	− 67,302.30	0.98^28^
	NM	8.46	–	1024.54	139.68	4.68	− 66,359.89	
Ba_2_TmNbO_6_	FM	8.50	8.42^29^	1015.58	141.66	4.62	− 68,267.06	0.99
	NM	–	–	1016.05	139.63	4.61	− 68,267.02	
Ba_2_SmNbO_6_	FM	8.51^49^	8.51	–	156.00	4.60	− 61,964.40	
		8.53^50^		1062.5	148.40	5.03	− 61,972.23	
Ba_2_LaNbO_6_	FM	8.71^51^	8.60^[Expt]^	–	130.00	–	− 58,096.40	0.96

To gain further insights on these oxide perovskites has been extended to establish the structural stability interms of their Goldschmidt tolerance factor (τ)^48^. This concept was usually introduced to assign the structural stability of perovskites from the ratio of the ionic radii and is exploited unanimously for this purpose. However, it describes the most probable ground state crystal structure. The value of tolerance factor significantly varies as, if the value of *τ* approaches to unity, means no geometrical strain is present and hence corresponds to perfectly ideal face centered cubic geometry with *Fm-3m* space group.

If *τ* deviates from unity means less chances of cubic phase to be form. If *τ* = 0.71–0.9, results in orthorhombic or rhombohedral structure. The predicted values of tolerance factors (*τ*) perfectly lies within the cubic range without any kind of structural distortions thus, signifies the face centered cubic geometry with *Fm-3m* space group.

### Second-order elastic constants and mechanical stability

Understanding the elastic properties of a material needs expensive efforts or we can say that they are quite tedious to measure them experimentally. But, theoretically speaking, several computational codes are so much reliable to figure out them with high accuracy *Wien2k* is one of them. Therefore, from the computer calculations, the elastic constants has been calculated using cubic elastic package by second order derivative (E″ (δ)) of polynomial fit (E = E(δ)) of energy vs. strains (δ) at zero strain (δ = 0) called energy approach^41^. However, the required lattice constant retrieved from the structural optimisations yields the better accuracy of elastic constants. The elastic constants correlates the elastic strength of a material towards the external loads. From the above structural findings, as both these materials Ba_2_ErNbO_6_ and Ba_2_TmNbO_6_ display cubic symmetry. Therefore, only three elastic constants viz; *C*_11_, *C*_!2_ and *C*_44_ are required to represent the elastic stability of these perovskites. Here, *C*_11_ stands for longitudinal compression defines the hardness/stiffness of the material, while *C*_12_ represents the transverse expansion inclined to Possion’s ratio and *C*_44_ is linked with the shear modulus. The procedure is simple while establishing them to the total energy as the function of lattice deformation within the restrictions of Born-stability criterion condition^52,53^ which is;

3 $$ C_{11} > 0,\quad C_{12} > 0\quad {\text{and}}\quad C_{44} > 0,\quad C_{11} {-}C_{12} > 0\quad {\text{and}}\quad C_{11} + 2C_{12} > 0 $$

Therefore, for Ba_2_ErNbO_6_ and Ba_2_TmNbO_6_ perovskites, the stability outlook is reflected from the estimated three elastic tensors enlisted in Table 2. Thus, the non-negative values indicates that both are elastically stable. The successful re-optimisation plots while calculating them (not shown here) also reflects the stability of these materials. In addition, the mechanical stability of these oxide perovskites has been keenly evaluated by consuming the different numerical values of elastic constants in several mathematical equations to figure out for their practical applications in industrial and technological perspective needs. These mechanical parameters have been elucidated under the following equations as;

4 $$ B_{V} = B_{G} = B = \frac{{(C_{11} + 2C_{12} )}}{3\,} $$

**Table 2:** Reported second-order elastic constants and mechanical stability of Ba_2_ErNbO_6_ and Ba_2_TmNbO_6_ perovskite at “0 GPa” and “0 K”.

Parameter	*C*_11_	*C*_12_	*C*_44_	*C″*	B	Y	B/G	ν	A	γ	Tm	E_coh_
Ba_2_ErNbO_6_	263.87	80.12	75.57	4.55	141.37	204.79	1.76	0.26	0.82	1.08	1901.37	6.29
Ba_2_TmNbO_6_	261.23	79.22	72.45	6.77	139.89	200.23	1.76	0.26	0.79	1.08	1887.88	6.27
Ba_2_SmNbO_6_^50^	270.77	90.23	82.45	7.78	150.40	193.77	1.99	0.28	0.92	–	–	6.38
Ba_2_LaNbO_6_^51^	265.9	68.20	58.50	9.78	134.00	183.50	1.85	0.28	–	–	2124.7	–

Shear modulus (G) as;

5 $$ G_{V} = \frac{{(C_{11} - C_{12} + 3C_{44} )}}{5\,};\quad G_{R} = \frac{{5(C_{11} - C_{12} )C_{44} }}{{4C_{44} + 3(C_{11} - C_{12} )}};\quad G = \frac{{G_{V} + G_{R} }}{2} $$

And, Young’s modulus (Y), Poisson’s ratio (υ) and Cauchy’s pressure are considered as,

6 $$ {\text{Y}} = \frac{{9{\text{BG}}}}{{3{\text{B}} + {\text{G}}}};\quad \upsilon = \frac{{3{\text{B}} - {\text{Y}}}}{{6{\text{B}}}};\quad {\text{C}}^{\prime\prime } = {\text{C}}_{{{12}}} - {\text{C}}_{{{44}}} $$

The first mechanical property evaluated from the set of elastic constants is bulk modulus (B) which explains the volumetric change of these materials upon the influence of external pressure. The calculated value of bulk modulus systems offer a decent value hence, explains the incompressible nature of these two perovskites. Another noteworthy property, called Young’s modulus of elasticity (Y) pronounces the stiffness of a material and principally displays a crucial role in understanding the structure of the material. From the Table 2 it is quite obvios that both these compouds have a good numerical value of Y defines high stiffness in character i.e.; the materials upon the external force will experience a small change in their shape. In the same way, the shear modulus (G) illustrates the transverse deformation. The mechanical stability has been further explored to see the ductile and brittle characteristics of both these materials. The various parameters among which Pugh’s ratio (B/G)^54^ primarily distinguishes the characteristic feature. As according to Pugh, a material is referred as ductile, if its values surpasses the limiting value (1.75) and brittle if it is beneath the same value. From the description, it is quite assured that these perovskites are categorised to be ductile as their B/G exceeds from the limiting value. Possion’s ratio (*v*)^55^ and Cauchy’s pressure (C″)^56^ discloses the ductile character also. Moreover, Possion’s ratio (*ν***)** measures the stretching of an object in one direction and shrinking in perpendicular. It is positive for the solids by satisfying the condition that B⁓G or B>>G. The negative value signifies the condition is reversed and the materials with negative Possion’s ratio are called “auxetic materials” or “auxetics”. Anisotropy (A)^57^ equal or greater than unity ensures materials are purely isotropic and anisotropic respectively. The recorded values favours the anisotropic behaviour thus, defines their properties in different directions. Further, the predicted value of Grüneisen parameter **(**γ) visualises the less involvement of phonon vibrations. In addition, melting temperature (Tm)^58^ describes the applicability of these oxides to retain their lattice structures over a wide range of temperatures. Finally, the expedition of cohesive energy (E_coh_) reveals the extention of bond strength and also signifies that large amount of energy is supplied to knock an atom from both these perovskite materials. A little bit information can be perceived as its large and positive value obviously explains the structural stability of perovskite structures.

### Phonon stability

**Figure 3:**
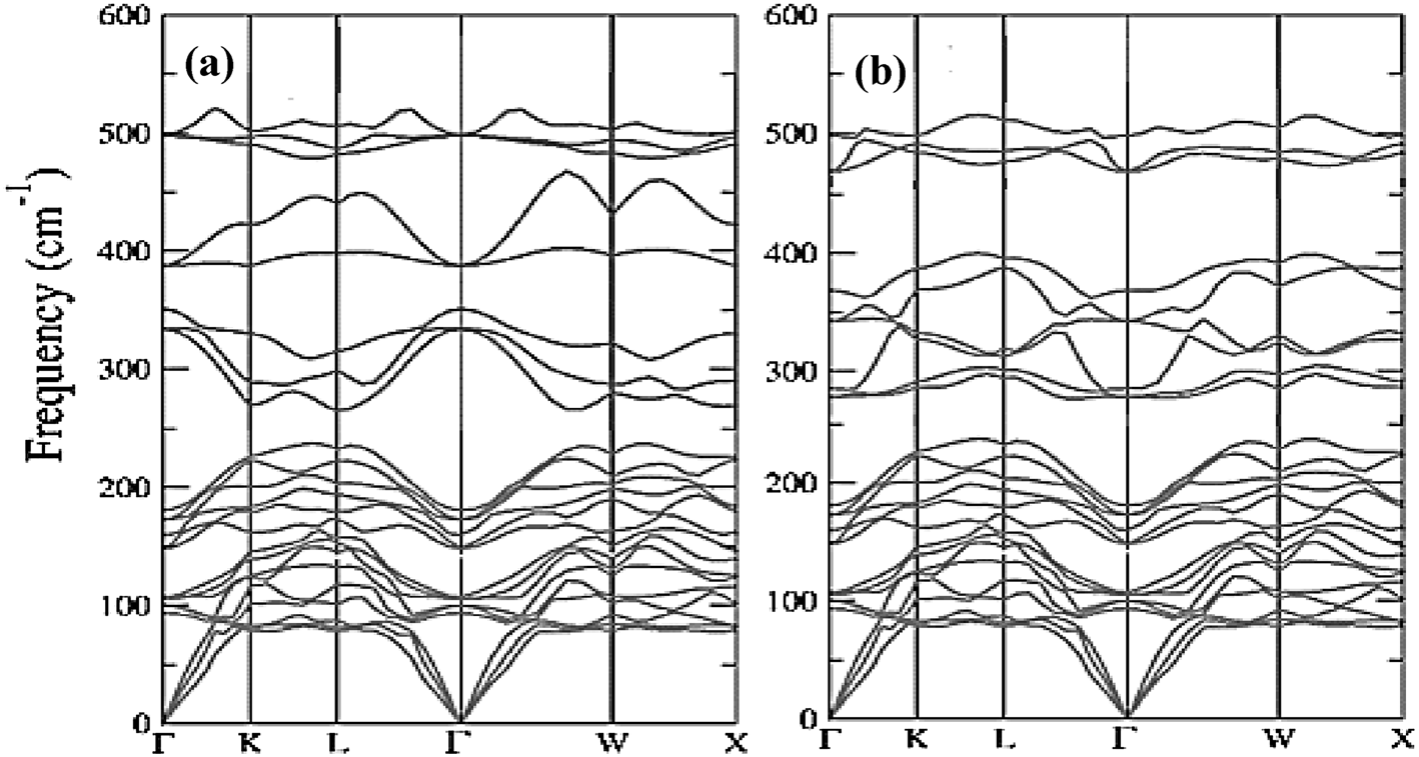
**(a**, **b)** Phonon dispersions along the high symmetric directions of Brillouin zone of (**a**) Ba_2_ErNbO_6_ and (**b**) Ba_2_TmNbO_6_ respectively.

Phonon dispersions are quite essential to perceive the dynamical context and vibrational Raman spectroscopy of materials^59^. In the present case, the full advantage of density functional perturbation theory (DFPT) as embedded in Quantum espresso^42^ has been established to forecast the dynamical stability within the primitive unit cells of Ba_2_ErNbO_6_ and Ba_2_TmNbO_6_ perovskites. To probe, within the high symmetry directions of the first Brillouin zone, the 30-phonon branches resulting from 10 constituent atoms inclusive of acoustic (three) and optical (twenty-seven) have been displayed in Fig. 3a,b. The absence of negative frequencies from their subsequent band dispersions legitimates the dynamical stability of these complex systems. Moreover, the frequency of highest optical branch is nearly recorded at 535.28 cm^−1^ and 530.18 cm^−1^ respectively.

### Spin-electronic properties

**Figure 4:**
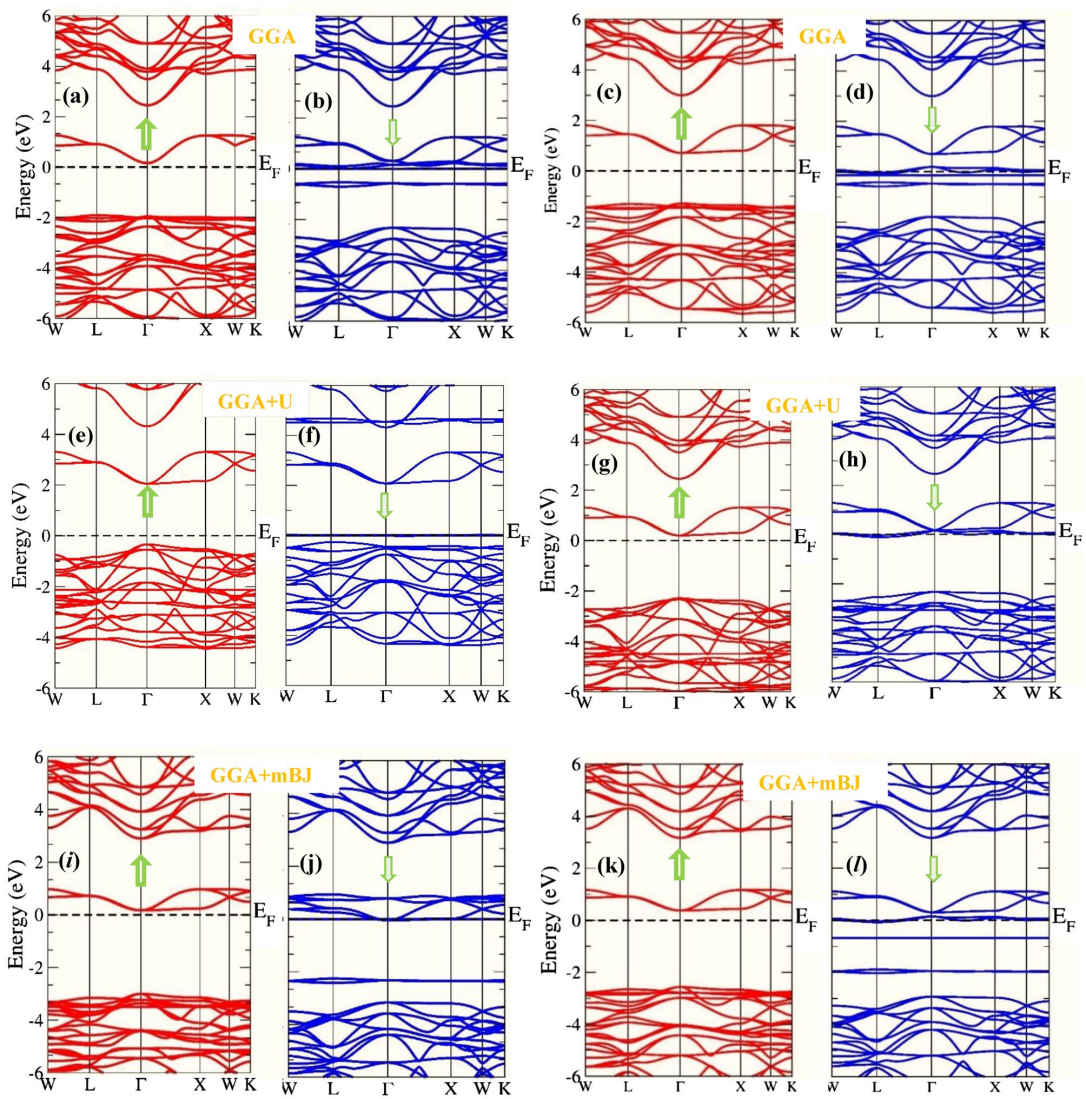
(**a**–**d**) DFT execution of band profiles of Ba_2_ErNbO_6_ and Ba_2_TmNbO_6_ wthin GGA where the arrows represents the possible spin-up and spin-dn orientations. (**e**–**h**) DFT execution of band profiles of Ba_2_ErNbO_6_ and Ba_2_TmNbO_6_ wthin GGA + U where the arrows represents the possible spin-up and spin-dn orientations. (**i**–**l**) DFT execution of band profiles of Ba_2_ErNbO_6_ and Ba_2_TmNbO_6_ wthin GGA + mBJ where the arrows represents the possible spin-up and spin-dn orientations.

**Figure 5:**
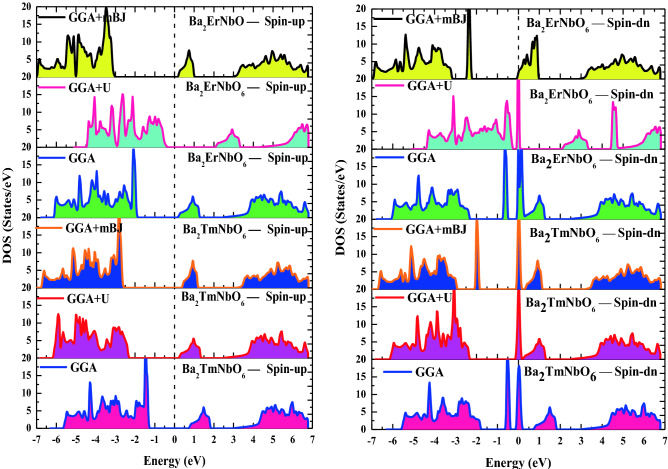
Spin polarised total density of states (DOS) in GGA, GGA + U and GGA + mBJ for Ba_2_ErNbO_6_ and Ba_2_TmNbO_6_ perovskites.

**Figure 6:**
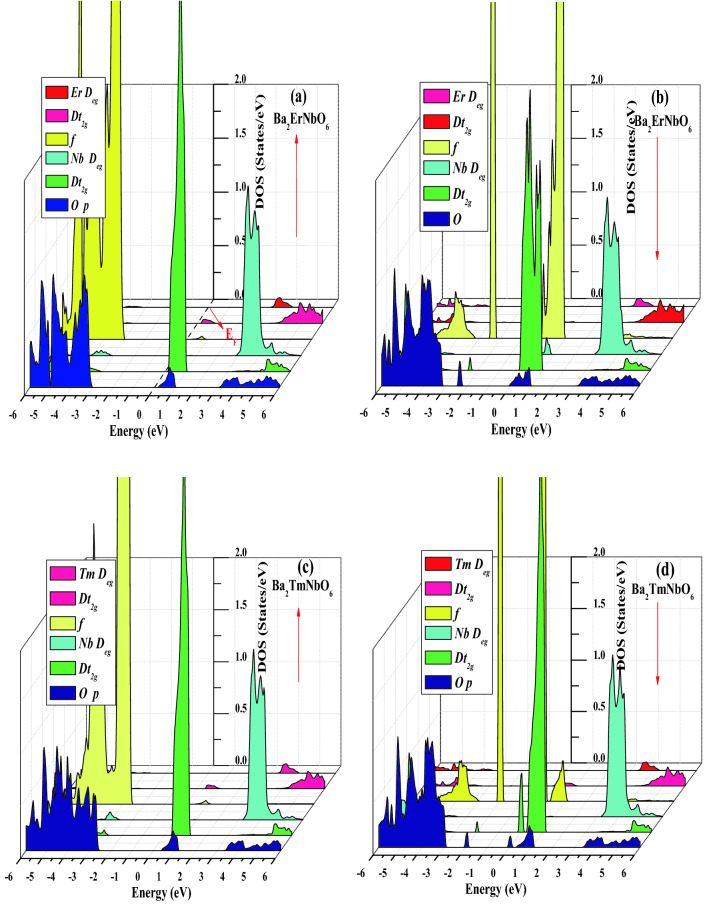
(**a**, **b**) Spin-polarised partial density of states (*pDOS*) of Ba_2_ErNbO6 via GGA + mBJ potential scheme, where zero at *x* axis designates the Fermi-Level (E_F_). (**c**, **d**) Spin-polarised partial density of states (*pDOS*) of Ba_2_TmNbO_6_ via GGA + mBJ potential scheme where, zero at *x* axis designates the Fermi-Level (E_F_).

The understanding of electronic structure is highly desired to define the possible applications of the material in dfferent areas of the scientific research. Since the energy band gap of the material characteristically describes its smart/spintronic/memory device applications. On the other hand, theoretical quantum descriptions decisively picturizes the nature of material interms of their electronic structures. Moreover, it is certified that materials can be classified on their electronic properties such as metals, insulators, semiconductors, spin-gapless semiconductors, half-metals etc. Herein, for the case of Ba_2_ErNbO_6_ and Ba_2_TmNbO_6_ double perovskites, the electronic structures has been executed by taking the advantage of relaxed lattice constants at the behest of their structural optimizations. The relaxation of these constants by passing them in GGA, GGA + U and along with the potential scheme of GGA + mBJ facilitates the essential properties interms of their electronic band profiles and associated density of states. These materials under the selected approximation schemes of GGA, GGA + U and incorporation of GGA + mBJ signatures the half-metallic character with the up-spin as semiconducting while the opposte dn-spin insights the character of metallic nature as evident from Fig. 4a–l. Seperatively, within the functional scheme of GGA the band gap perceived for both the systems in the up-spin orientation comes out to be 2.07 eV and 2.00 eV respectively. But consistent with this, it is well reported that GGA is not sufficient for *d/f* systems to execute the interpretation of electronic structures particularly the reason of severe underestimation of band gap problem makes the GGA ineffective. Meanwhile, the calibration of GGA + U is therefore, a next choice to refine their electronic structures in a precise way. Although the sophisticated potential is extremely viable in resolving the electronic structures within underestimated regimes and provides a well reasonable to predict the electronic structure of highly correlated systems, but the method is sem-empirical. However, the band gap calculated by the approximation scheme in spin-up orientation for both these is recorded to be 2.35 eV and 2.32 eV respectively. Therefore, the presence of *d/f* electrons invokes us to employ a new strategy known as modified Becke Johnson potential which is much favorable among all the approximation methods to exploit the intrinsic characteristics of electronic structure of these materials. Thus, the implementation of GGA + mBJ potential also describes the half-metallic nature is maintained within these oxide systems prevailing the band gap of 3.00 eV and 2.94 eV respectively. The theoretical background demonstration of displaying the corresponding half-metallic nature within their intrinsic lattice structures can be briefly explained with the help of well-known Crystal Field Splitting (CFT) theory. The structural arrangement of atoms by assuming the polyhedra inbetween Er, Tm and Nb atoms. These atoms are specifically enclosed within their cages surrounded by the O atoms. The interplay of Coulomb interaction in between them is quite responsible to decrease the degeneracy of Er/Tm *f*-orbital into *f*_a1g_, f_t1g_, *f*_t2g_ subsets and Nb *d*-orbital splits into triplet *d*_t2g_ (*d*xy, *d*yz, *d*zx) and doublet *d*eg (*dx*^2^ − *y*^2^, *dz*^2^) sets. The intake capacity of *f*_a1g_ contains only 2 electrons. On the other hand, the *d*_t2g_ accommodates 6 electrons (3↑ + 3↓) while *d*eg takes 4 electrons (2↑ + 2↓) followed by Pauli’s exclusion principle. The filling of electrons with respect to their nominal oxidation states and amount of splitting to form low or high complex depend upon the ligand interacting whether weak or strong. As in their octahedral compartments the six oxygen atoms are interacting with the central atoms enclosed within their cage like structures creates a weak field environment. Cutting in short, the filling of electrons will be in accordance with the Hund’s rule thus forming the high spin state which accordingly demonstrates the half-metallic electronic structures of these perovskites. Further, physical interpretation of electronic band structures can be analysed from their spin-polarised density of states (TDOS) and their specified partial density of states (pDOS). The refined total density of states labelled in Fig. 5 and their accompanied partial density of states within GGA + mBJ scheme as portrayed in Fig. 6a–d which also directs the half-metallic character of these perovskites. As, aforementioned, the overall unsymmetrical band profiles along with resulting density of states of Ba_2_ErNbO_6_ and Ba_2_TmNbO_6_ from their corresponding spin channels (spin-up/spin-dn) in different exchange correlation schemes favours the spin polarisation effect has a predominant role in demonstrating the electronic structure of these materials which can lead the spin-polarised currents. Moreover, pseudo gap within their band structures is formed which is basically a true gap where the possibility of energy state is not allowed. The main occurrence of pseudo gap in both the compounds is due to the fact of crystal field splitting. In addition, possible indirect-exchange mechanism within these systems is also responsible to display the pseudo gap in these compounds. On viewing, the band gap trend from Er to Tm upon GGA + mBJ scheme reflects decreasing scenerio. The possible reason is attributed due to direct consequence of increasing the lattice constant which intern decreases the band gap of these materials. Another reason is due to increase in the atomic size, which is inversely proportional to the band gap of material. Therefore, the presence of gap in the spin-up and absence of gap within the spin-dn orientation is responsible to depict the well-known half-metallic nature within these perovskite systems.

### Magnetic properties

Magnetism an intrinsic property comprehensively related to the magnetic materials. Due to the complex nature and flexibility of two magnetic sites (BB′) within the generic formula unit of A_2_BB’X_6_ offers a decent magnetism. The two atomic sites not only possesses the essential quantity of magnetism within their own lattice structures but they are also quite adaptive in transforming the shape of the perovskite structure. In this present piece of investigation, the rare earth-based Ba_2_ErNbO_6_ and Ba_2_TmNbO_6_ materials are of special kind of interest in which magnetism is certainly inherited within their own lattice structures thus, aids their facial route towards spintronic applications. The early supportive information of occurrence of intrinsic magnetism can be forecasted from their resulting unsymmetrical behavour of corresponding two-dimensional band structures. To analyse magnetism of these representative oxide perovskites which were thoroughly studied under the simulation process of *Wien2k* upon GGA, GGA + U along with the mBJ functional schemes as shown in Table 3. On employing the computation of these approximation schemes, it is foreseen that the incorporation of total spin magnetic moment rather than orbital magnetic moment within Ba_2_ (Er/Tm) NbO_6_ perovskites has been found to 3 µ_B/_*f*.u and 2 µ_B/_*f*.u respectively which is well consistent with the prime reported values of others. However, the individual contributions of distinct atoms taking part within their corresponding lattice structures has been keenly analysed. From the Table 3 it is clear that, the maximum magnetism is mostly governed from Er in case of Ba_2_ErNbO_6_ and Tm in Ba_2_TmNbO_6_ atoms because the energy states Er-*f* and Tm-*f* are mostly pinned around the reference level. On the other hand, the magnetism of other individual atoms viz; Ba, Nb and O in both the structural cases are very feeble because of their paramagnetic behaviour. Paramagnetic compounds have symmetric band structures hence they achieve their magnetism when they are kept in externally applied magnetic field^60,61^. The positive and negative value of magnetic moment tells the ferromagnetic and antiferromagnetic/ferrimagnetic interactions among the various constituents involved within their lattice structures. Infact, the origin of magnetism within these prescribed oxide compounds is attributed due to indirect exchange interaction between the adjacent Er atoms via nonmagnetic anion O (Er-O-Er) and O (Tm-O-Tm). The resulting exchange mechanisms are double-exchange and super exchange. Most often, ferromagnetic leads to the double exchange and super exchange leads favours anti-ferromagnetism but sometimes the situation is reverse. However, Zener^62^ reported that the sufficient separation of magnetic atoms and conduction electrons present in a crystal favour the ferromagnetic interactions, which is due to the immediate transfer of electrons from Er/Tm to O and then from O to nearest Er/Tm in our case. The amount of spin magnetic moment from their periodic lattice structures creates a better tendency to display their applications in spintronics, magnonics etc. A little bit over view on spintronics (spin transport electronics) generally describes a new field of science which harnesses the spin degree of freedom in addition to its fundamental charge of electron. The requirement of spin signal is faster and thus, produces less heat dissipation when it comes into play for transportation of data for longer distances and is specifically used to embed or compress large storage of data in a smaller area. However, it is believed that “Race track memory” a three-dimensional memory has even degree of storage density can replace hard disks in the near future.

**Table 3:** Spin magnetic moment of Ba_2_Er/TmNbO_6_ perovskites.

Parameter	Magnetic moment (*μ*_B/_ *f*.*u*)						Gap (eV)
	Ba	Er/Tm	Nb	O	Inter	Total	
**Ba**_**2**_**ErNbO**_**6**_							
GGA	0.00	2.85	− 0.01	0.02	0.04	3.00	2.07
GGA + U	0.00	2.92	0.00	0.06	0.02	3.00	2.35
mBJ	0.00	2.99	− 0.02	0.00	0.02	2.99	3.00
**Ba**_**2**_**TmNbO**_**6**_							
GGA	0.00	1.84	− 0.01	0.02	0.05	2.00	2.00
GGA + U	0.00	1.91	− 0.06	0.02	0.01	1.98	2.32
mBJ	0.00	1.92	− 0.04	0.02	0.00	2.00	2.94

### Electronic-charge density

**Figure 7:**
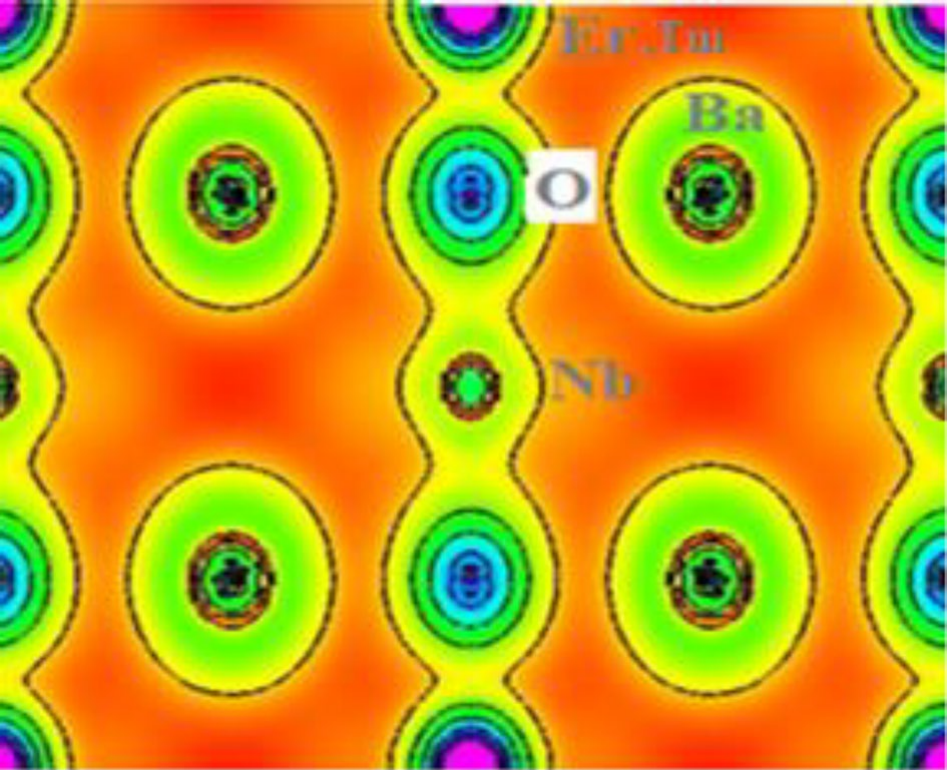
Spatial charge density description of Ba_2_Er/TmNbO_6_ oxide perovskites.

The description of chemical properties of an atom can be explained by the electronic structure and electronic configuration. As according to Pauling, a chemical bond exists in between two atoms when the bonding force between them is very strong. The chemical bonds can be discussed interms of ionic, covalent and metallic bond. Ionic bonds are also called electrovalent bonds in which the transfer of electrons from one atom to another takes place and these bonds can be understood without quantum theory. In contrast to covalent which gets formed by the mutual sharing of electrons thus can be analysed by same theory. Moreover, the various metallic bonds existing are hydrogen bonds, dipole and London bonds which are occasionally defined by Wander vall’s forces. In consistent with this, and on exploring these perovskites interms of their chemical stabilities, we have tried to explain the nature of bonding characteristics among the neighbouring atoms of Ba_2_ (Er/Tm) NbO_6_ with the help of charge density plots described in [111] plane as shown in Fig. 7. Here, we have taken an assumption to figure out the bonding characteristics. The nature of charge is properly identified in between the different constituents of the atoms. The linkage between Ba and O is properly ionic in character because the electron cloud of both the atoms are completely spherical within itself and takes the electrons within own. However, the nature of bonding between the O and Nb is mostly covalent in character. The ionic nature character can be rectified due to fact of partially *d* subshells of Nb transition metal. Therefore, the overall bonding characteristics of these perovskite systems poses its polar covalent nature i.e.; the admixture of covalent and ionic bonding is linked within these perovskites.

### Thermoelectric properties

**Figure 8:**
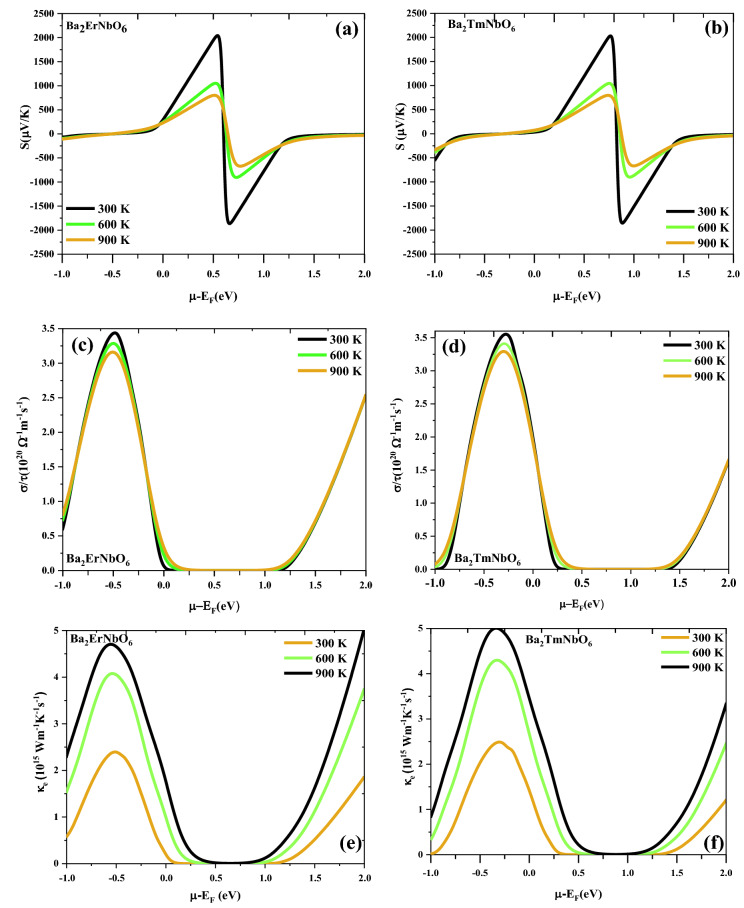

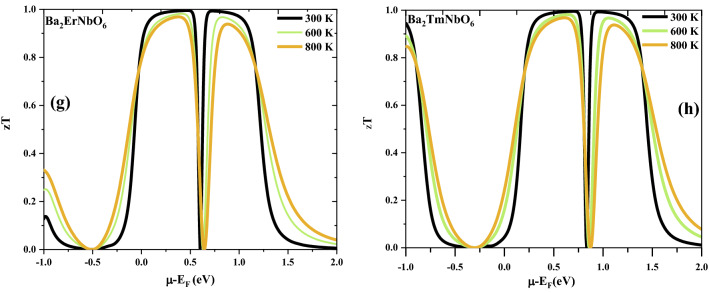
(**a**–**h**) Variation of (**a**, **b**) Seebeck coefficient (S), (**c**, **d**) electrical conductivity (σ/τ), (**e**, **f**) electronic thermal conductivity (κ_e_), (**g**, **h**) Figure of merit (zT) versus chemical potential of Ba_2_ErNbO_6_ and Ba_2_TmNbO_6_. Different colors are used to distinguish the temperature: Black-300 K; Green-600 K; and Wine-900 K.

Thermoelectric coefficients has been calculated by using the semi-classical Boltzmann transport theory^43,44^ as merged in BoltzTraP program which provides numerically stable and efficient method of obtaining analytical representations within the constraints of relaxation time constant (τ). The proper estimation of thermoelectric efficiency of Ba_2_ErNbO_6_ and Ba_2_TmNbO_6_ materials has been achieved by investigating the transport parameters like Seebeck coefficient (S), electrical conductivity (σ/τ), thermal conductivity (κ) and the figure of merit (zT) as a function of the chemical potential (µ) at three distinct temperatures (300, 600, 900) K. The positive and negative values of µ defines the *n* and *p*-type regions respectively. From the understandings of the Seebeck coefficient (S) of these two perovskite systems as labelled in Fig. 8a,b. The Seebeck coefficient in the entire range of the chemical potential, picturizes the prominent peaks and valleys. It is clearly visualized that there are alternatively high-intensity peaks for both positive as well as negative potentials at 300 K and these peaks decreases as temperature rises. The reason behind is because bound electrons get excited by acquiring thermal energy, generate electron–hole pairs. The most prominent peaks are in the range of 0 to 1 eV as the bands are less dispersive with the forbidden region around Fermi level, thereby fewer charge carriers are around in this range. The maximum value of S is 2100 μV/K for Ba_2_ErNbO_6_ and 2000 μV/K for Ba_2_TmNbO_6_ at 300 K respectively. On comparing the results, it can concluded that Ba_2_ErNbO_6_ shows a better Seebeck coefficient because of the presence of a larger bandgap and highly correlated nature of localised *f-*bands. In addition, the justification of the above statement can be made more interesting as experimentally noted by Gambino et al.^63^ that many rare earth compounds in which the *f-*level is near to the Fermi energy generates large Seebeck coefficients. For these materials, the *f*-bands are narrow resulting a very high density of states near the Fermi level. Subsequently, on studying the Seebeck coefficient of these materials in response to temperature can offer a distinct voltages in their corresponding spin-up as well as spin-dn channels as these materials are half-metallics. By considering the metallic channel induces the Seebeck voltage around 10 µV/K as metals are poor thermoelectric materials due to their higher values of thermal conductivity which intern reduces the square of Seebeck coefficient hence are not capable of featuring good thermoelectrics. In contrast to opposite semiconducting spin channel significantly adopts a large value of Seebeck coefficient thus contradicts the distinct distribution of Seebeck in different spin channels. This special kind of aspect makes the half-metallic unit cell to behave intrinsically as thermocouple. So, when this thermocouple is subjected to temperature gradient it generates driving powers in their corresponding spin directions thus giving rise to Spin Seebeck effect^64^. Next, we have tried to see the variation of electrical conductivity over the relaxation time (σ/τ) estimated at the three different temperatures as a function of chemical potential. The behaviour of electrical conductivity of these two perovskite compounds is descripted in Fig. 8c,d which falls to zero within a range of almost 0–1.23 eV for Ba_2_ErNbO_6_ and 0.25–1.3 eV for Ba_2_TmNbO_6_ of the chemical potential. The reason summarises that the absence of energy bands around the Fermi level making the area desolate of charge carriers and hence the conductivity vanishes around μ-E_F_ = 0. Beyond this range and also with rise in temperature, electrons make the transition from valence band as the certain energy states of electrons are filled at T = 0 K becomes empty to the conduction band thereby increases the conductivity on both sides. As that of this, the other thermoelectric property called, electronic thermal conductivity (κ_e_) of these compounds has also been presented in Fig. 8e,f. The electronic thermal conductivity increasing with increase in temperature. The reason may be attributed as the electrons get sufficient thermal energy and switches for the conduction. Finally, the intrinsic parameters have been collected to reveal the zT value of Ba_2_ErNbO_6_ and Ba_2_TmNbO_6_ as shown in Fig. 8g,h. The occurrence of prominent peaks explains their zT value almost equal to unity (1) inclines their facial route towards energy harvesting, nanoengineering, as well as in radioisotope generator applications (RTG, s) where the decay energy of radioactive substance is acted upon the series of thermocouples joined in cascaded form to transform the energy back into usable electrical form by the principle of Seebeck effect.

### Conclusions and outlook

To conclude, with the help of exploited density functional theory, an extensive over view on rare earth-based perovskites Ba_2_ErNbO_6_ and Ba_2_TmNbO_6_ influenced by their electronic structures has been summed up. Primiraly, the total ground state energies and reasonable values of tolerance factor better yields the required structural stability of these given compounds. The positive value of second order elastic constants and their derived mechanical parameters justifies the ductile features acertain them. In associate with this, the cohesive energies of these layered structures gaurantess the extension of bond stabity. Also, the absence of phonon frequeny vibrations absolutely defines the dynamical stabity. The various calibrated functionals over the exchange correlation signatures while defining their electronic structure claims the well-known half-metallic nature. The band-gap while shifting from Er to Tm within the functional scheme of GGA + mBJ shows decreasing trend with the explanation of increasing lattice constant. Moreover, the information retrieved from their resulting band structures forecasts the intrinsic magnetic character of (3, 2) μB thus, certifies the half-metallicity is ensured within these considered oxide systems. Also, the estimated values of Seebeck coefficient (S) on probing the transport parameters are quite satisfactorily and a decent value of figure of merit (*zT*) equal to unity along the selected chemical potential at different temperatures potentially felicitates their superior applicability in power technological and nanoenginerring applications. Such intriguing properties may also register them in the category of multifunctional or smart materials.

## Data Availability

The data would be available from the corresponding author on a reasonable request.
